# A Synergistic Approach Using Photoacoustic Spectroscopy and AI-Based Image Analysis for Post-Harvest Quality Assessment of Conference Pears

**DOI:** 10.3390/molecules30112431

**Published:** 2025-06-01

**Authors:** Mioara Petrus, Cristina Popa, Ana Maria Bratu, Vasile Bercu, Leonard Gebac, Delia-Mihaela Mihai, Ana-Cornelia Butcaru, Florin Stanica, Ruxandra Gogot

**Affiliations:** 1 Laser Department, National Institute for Laser, Plasma and Radiation Physics, 409 Atomistilor St., P.O. Box MG 36, 077125 Magurele, Romania; cristina.popa@inflpr.ro (C.P.); ana.magureanu@inflpr.ro (A.M.B.); 2 Faculty of Physics, University of Bucharest, 405, Atomistilor Str., 077125 Magurele, Romania; vasile.bercu@unibuc.ro (V.B.); leonard.gebac@unibuc.ro (L.G.); delia-mihaela.mihai@s.unibuc.ro (D.-M.M.); 3 Research Center for Studies of Food Quality and Agricultural Products, University of Agronomic Sciences and Veterinary Medicine, 59, Marasti Blvd., 011464 Bucharest, Romania; ana.butcaru@qlab.usamv.ro; 4 Faculty of Horticulture, University of Agronomic Sciences and Veterinary Medicine, 59, Marasti Blvd., 011464 Bucharest, Romania; florin.stanica@usamv.ro (F.S.); ruxandra_gogot@yahoo.com (R.G.)

**Keywords:** ethylene, ethanol, ammonia, CO_2_ laser photoacoustic spectroscopy, post-harvest quality, fruit senescence, artificial intelligence, convolutional neural network

## Abstract

This study presents a non-invasive approach to monitoring post-harvest fruit quality by applying CO_2_ laser photoacoustic spectroscopy (CO_2_LPAS) to study the respiration of “Conference” pears from local and commercially stored (supermarket) sources. Concentrations of ethylene (C_2_H_4_), ethanol (C_2_H_6_O), and ammonia (NH_3_) were continuously monitored under shelf-life conditions. Our results reveal that ethylene emission peaks earlier in supermarket pears, likely due to post-harvest treatments, while ethanol accumulates over time, indicating fermentation-related deterioration. Significantly, ammonia levels increased during the late stages of senescence, suggesting its potential role as a novel biomarker for fruit degradation. The application of CO_2_LPAS enabled highly sensitive, real-time detection of trace gases without damaging the fruit, offering a powerful alternative to traditional monitoring methods. Additionally, artificial intelligence (AI) models, particularly convolutional neural networks (CNNs), were explored to enhance data interpretation, enabling early detection of ripening and spoilage patterns through volatile compound profiling. This study advances our understanding of post-harvest physiological processes and proposes new strategies for improving storage and distribution practices for climacteric fruits.

## 1. Introduction

Fruit respiration is a key physiological process influencing post-harvest quality and shelf life [1]. Volatile organic compounds (VOCs), such as ethylene and ethanol, play crucial roles in ripening and spoilage [2]. Traditional gas analysis methods often lack the required sensitivity for real-time monitoring, making laser-based techniques, such as photoacoustic spectroscopy (PAS), highly valuable. PAS enables non-invasive, high-precision detection of trace gases, providing insights into metabolic changes during fruit storage [3,4].

Ensuring fruit quality and shelf life in the post-harvest period is an important aspect of the agri-food chain, directly influencing freshness, nutritional value, and consumer acceptance [1,5]. In this context, advanced methods for analyzing fruit respiration provide valuable insights into physiological processes and help identify differences between fruits from distinct sources [6,7,8].

The “Conference” pear (*Pyrus communis* L.) is one of the most widely recognized pear cultivars, valued for its smooth, creamy texture and sweet, slightly aromatic taste. It is highly regarded both as a table fruit and for use in various culinary applications [9]. As a climacteric fruit, Conference pears exhibit a characteristic increase in ethylene production and respiration rate after harvest, directly impacting their ripening and storage behavior. Their relatively short harvesting period (typically 4–6 months) and sensitivity to storage conditions necessitate effective monitoring to optimize post-harvest management. Factors such as temperature, humidity, and post-harvest treatments significantly influence fruit respiration, affecting ripening and spoilage [10,11].

Post-harvest quality assessment is particularly important for climacteric fruits like pears. Traditional methods for assessing fruit ripeness and quality, such as firmness measurements, color analysis, and sensory evaluation, often rely on physical and chemical analyses [12]. While effective, these methods are time-consuming and often invasive, leading to the need for more efficient, real-time techniques. In recent years, non-invasive methods like gas chromatography (GC), mass spectrometry (MS), and electronic noses have been applied to monitor volatile compounds [13,14,15]. While GC and MS provide high sensitivity and accuracy, they require sample preparation and are labor-intensive. Electronic noses offer real-time monitoring but often lack the specificity and sensitivity required for precise measurements [16,17,18].

In contrast, CO_2_ laser photoacoustic spectroscopy (CO_2_LPAS) offers a promising non-invasive alternative for real-time monitoring of respiration gases [19,20]. This technique can measure the concentrations of volatile compounds like ethylene, ethanol, and ammonia with high sensitivity and precision, making it an ideal tool for assessing physiological changes in fruits [21,22]. Although previous studies have employed CO_2_LPAS to analyze various fruits, including apples and bananas, there is limited research on its application to Conference pears, particularly for ammonia as a potential biomarker for fruit degradation.

Dedicated software analysis used in spectroscopy shows real advantages [23]. The integration of AI with techniques such as CO_2_LPAS enables efficient analysis of volatile compounds emitted by fruits, such as ethylene, ethanol, and ammonia, providing valuable insights into post-harvest physiological processes [24]. These data reflect physiological changes such as ripening and degradation, and machine learning models, including Random Forest and Support Vector Machines (SVMs), can be trained to classify fruits based on condition or to predict quality evolution over time. Convolutional Neural Networks (CNNs), in particular, are effective in extracting visual features from fruit images for classification tasks and show high accuracy in quality assessment pipelines [25]. Recent work has also demonstrated the importance of selecting appropriate network architectures, implementing dropout regularization, and using data augmentation strategies (e.g., image flipping, rotation, brightness normalization) to enhance generalization performance and reduce overfitting [26,27]. In this study, we explore CNNs as a non-invasive tool to estimate pear ripening stages from visual data and correlate them with gas emission patterns, with the goal of developing an integrated, AI-driven post-harvest quality monitoring system. This study aims to compare the respiration dynamics of “Conference” pears by analyzing their VOC emissions. The novelty of this research lies in applying PAS for detailed gas profiling, offering potential improvements in quality control and storage strategies. It also investigates ammonia’s role as a potential biomarker for fruit degradation, an area that has not been extensively explored in the literature. Furthermore, we develop and train a CNN model to classify ripening stages based on VOC emission patterns, offering improved accuracy for real-time quality assessment and decision-making in post-harvest management.

## 2. Results

This section presents this study’s findings on volatile gas emissions from “Conference” pears’, focusing on ethylene (C_2_H_4_), ethanol (C_2_H_6_O), and ammonia (NH_3_) concentrations and their relationship to pear quality during shelf life.

### 2.1. Ethylene, Ethanol, and Ammonia Emissions

**Figure 1:**
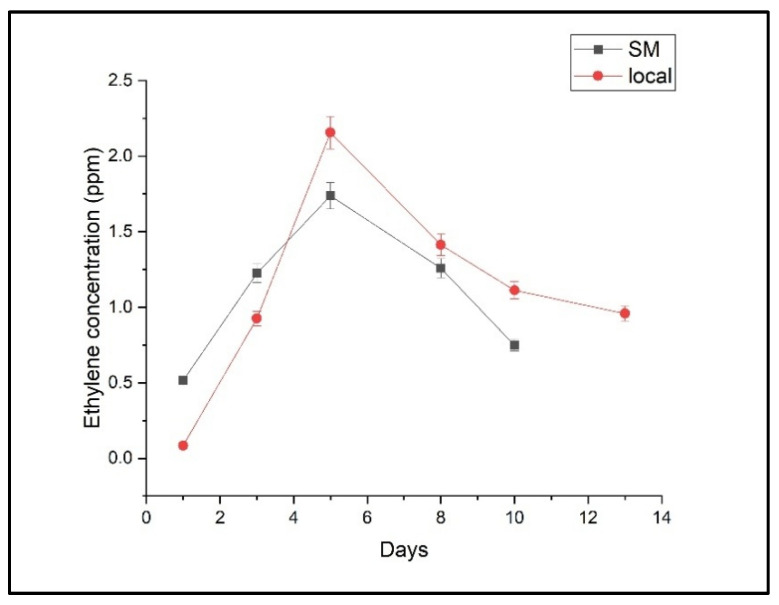
Evolution of ethylene concentration during shelf-life measurements in the two batches of “Conference” pears (local and supermarket). Data represent mean values ± 95% confidence intervals (*n* = 3 pears per time point).

**Figure 2:**
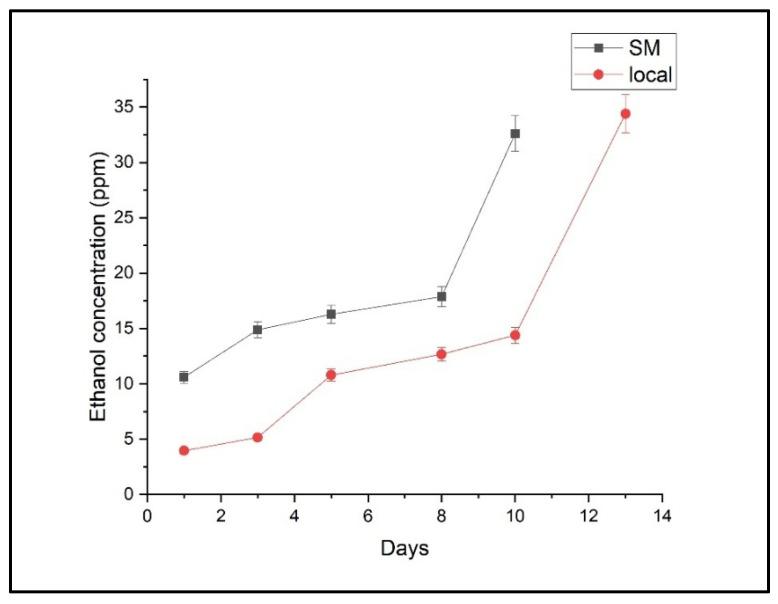
Evolution of ethanol concentration during shelf-life measurements in the two batches of “Conference” pears (local and supermarket). Data represent mean values ± 95% confidence intervals (*n* = 3 pears per time point).

**Figure 3:**
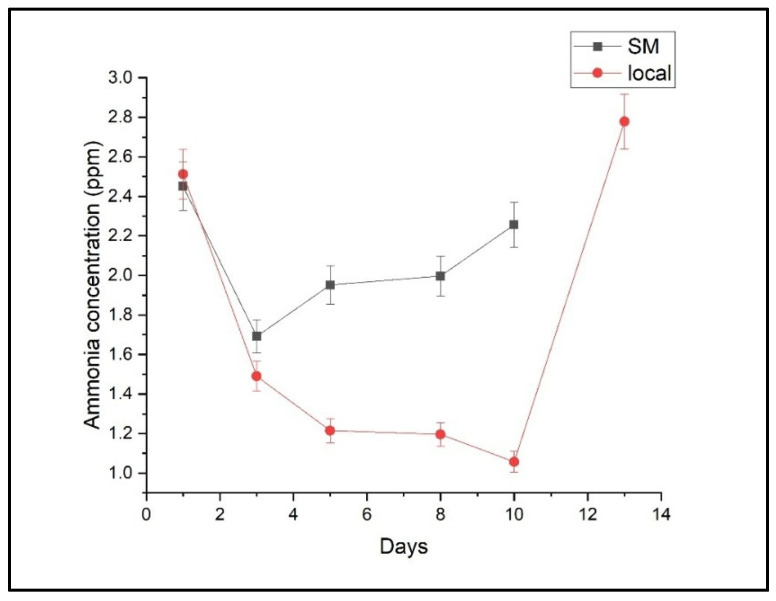
Evolution of ammonia concentration during shelf-life measurements in the two batches of “Conference” pears (local and supermarket). Data represent mean values ± 95% confidence intervals (*n* = 3 pears per time point).

The temporal evolution of ethylene, ethanol, and ammonia concentrations for both the local and supermarket batches of Conference pears is depicted in Figure 1, Figure 2 and Figure 3, respectively.

Ethylene concentrations exhibited a similar trend in both batches, with an initial increase followed by a peak around days 5–6, indicating the onset of full ripening. Specifically, the local batch reached a maximum ethylene concentration of 2.157 ± 0.678 ppm, while the supermarket batch peaked at 1.738 ± 0.478 ppm. The slightly earlier peak observed in supermarket pears suggests a potentially accelerated ripening process, possibly influenced by post-harvest treatments.

Ethanol concentrations generally increased over time in both batches, demonstrating a progressive accumulation of this volatile compound. A sharp rise in ethanol was observed after day 8 in supermarket pears and after day 10 in local pears, which likely signifies the onset of fermentation-related deterioration. At the end of the shelf-life storage, ethanol concentration was 34.406 ± 11.614 ppm for the local batch and 32.612 ± 8.368 ppm for the supermarket batch.

Ammonia concentrations showed a distinct pattern, with an initial decrease followed by a significant increase after days 8–10. This late-stage increase in ammonia suggests a potential association with metabolic degradation during senescence, positioning it as a possible biomarker for fruit deterioration. The maximum ammonia concentration from the local batch was 2.778 ± 0.744 ppm, and the maximum concentration from the supermarket batch was 2.451 ± 0.292 ppm.

To further explore the role of ammonia as a potential marker of fruit deterioration, we analyzed the time at which ammonia concentration began to increase in pear batches. A notable rise in ammonia concentration was observed in the local batch after day 10, coinciding with visible signs of fruit senescence such as softening and skin wrinkling. A similar trend appeared slightly earlier in the supermarket batch, around day 8. This suggests that the onset of ammonia accumulation may be linked to the degradation of amino acids and proteins during advanced ripening stages. The temporal correlation between ammonia increases and ethanol accumulation further supports the hypothesis that ammonia could be an early biochemical indicator of shelf-life decline. These findings highlight ammonia monitoring as a promising tool for real-time assessment of fruit freshness in post-harvest management.

To assess the magnitude of differences in gas concentrations between local and supermarket environments, Cohen’s *d* was calculated for ethylene, ethanol, and ammonia according to Equation (1). Cohen’s *d* is a standardized measure of effect size, indicating the magnitude of the difference between two means. Generally, a Cohen’s *d* of 0.2 is considered a “small” effect, 0.5 a “moderate” effect, and 0.8 a “large” effect.

(1) $$d=\frac{{\bar{X}}_{1}-{\bar{X}}_{2}}{s_{p}}$$

where $s_{p}$ is the pooled standard deviation and was calculated according to Equation (2):

(2) $$s_{p}=\sqrt{\frac{\left(s_{1}^{2}-s_{2}^{2}\right)}{2}}$$

For ethylene, a Cohen’s *d* of approximately 0.72 was observed, indicating a medium to large effect size. This suggests a notable difference in ethylene concentrations between the two environments, with local environments exhibiting higher mean levels (2.157 ppm, SD = 0.678) compared to supermarkets (1.738 ppm, SD = 0.478). In contrast, ethanol concentrations showed a small effect size, with a Cohen’s *d* of approximately 0.18. This minimal difference suggests that the environment type had a negligible impact on ethanol levels (local: 34.406 ppm, SD = 11.614; supermarket: 32.612 ppm, SD = 8.368). Finally, for ammonia, a Cohen’s *d* of approximately 0.58 was calculated, representing a moderate effect size. This indicates a perceptible difference, with local environments generally presenting slightly higher ammonia concentrations (2.778 ppm, SD = 0.744) than supermarkets (2.451 ppm, SD = 0.292).

### 2.2. Comparative Analysis of Storage Effects

**Figure 4:**
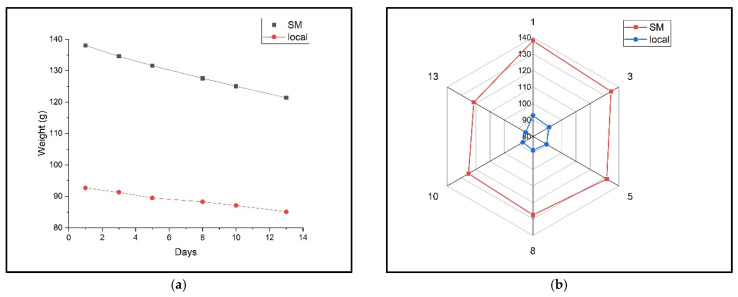
Analysis of the weight of the two batches of “Conference” pears, local and supermarket: (**a**) variation in the weight of the two batches of pears during the measurements; (**b**) the weight profile.

The change in pear weight during the measurement period is presented in Figure 4. This provides an indirect measure of water loss and overall deterioration. Supermarket pears showed a faster weight loss rate than the local batch, indicating a more rapid maturation and deterioration process. This observation aligns with the differences in ethylene and ethanol production, suggesting that post-harvest handling and storage conditions influence the metabolic processes. The local batch exhibited a lower average weight (92.66 g) than the supermarket batch (138 g) at the beginning of the experiment, which the authors attribute to drought conditions.

### 2.3. Statistical Analysis

**Figure 5:**
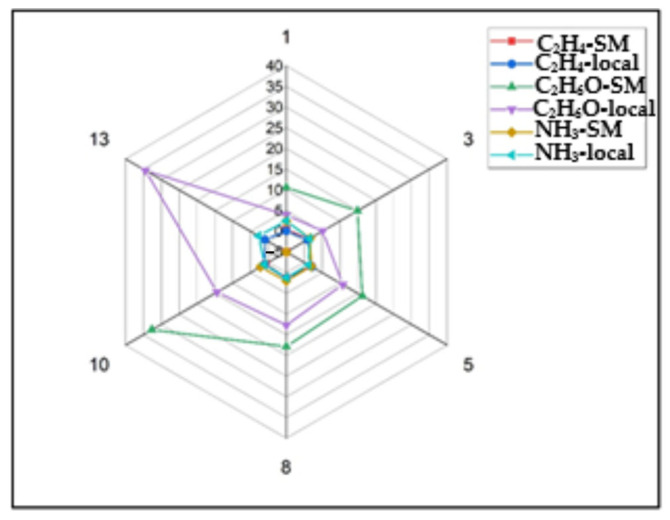
Radar profile for the gas concentration (ethylene—C_2_H_4_, ammonia—NH_3_, and ethanol—C_2_H_6_O) of the two batches of “Conference pears”: local and SM—supermarket.

Statistical analyses were performed to compare gas concentrations between the two pear batches and to explore the relationships between the different gases. Figure 5 shows the radar profile of the two batches of “Conference pears” according to the weight evolution during the measurement period, and supermarket pears show higher ethanol levels, possibly due to longer storage.

Pearson correlation coefficients were calculated to assess the relationships between ethylene, ethanol, and ammonia concentrations (Table 1). Strong positive correlations were observed between all gas pairs, particularly between ethylene and ethanol (*r* = 0.82, *p* < 0.001), suggesting that these gases are co-produced during ripening and spoilage. The correlation between ethylene and ammonia was 0.65 (*p* = 0.003), and the correlation between ethanol and ammonia was 0.71 (*p* < 0.001).

Two-way ANOVA was conducted to evaluate the effects of “Batch” (local vs. supermarket) and “Day” on gas concentrations. The null hypothesis (H0) was that there is no significant difference in gas concentrations between batches, while the alternative hypothesis (H1) stated that significant differences exist.

For ethylene, with results presented in Table 2, both “Batch” and “Day” had a statistically significant effect on ethylene levels (*p* < 0.001), and the interaction between “Batch” and “Day” was also significant (*p* = 0.02). This significant interaction indicates that the difference in ethylene levels between the two batches changed over time.

Table 3 presents the analysis for ethanol, similar to ethylene, both “Batch” and “Day” significantly affected ethanol concentrations (*p* < 0.001), and the interaction was also significant (*p* = 0.002). Specifically, supermarket pears had significantly higher ethanol levels than local pears, and ethanol concentrations increased more sharply over time in supermarket pears.

For ammonia, the “Day” factor had a significant effect on ammonia concentration (*p* = 0.001), indicating that ammonia levels changed significantly over time (see Table 4). However, there was no significant difference in ammonia levels between the local and supermarket batches (*p* = 0.18), and the interaction between “Batch” and “Day” was not significant (*p* = 0.41). This suggests that ammonia trends were similar in both batches, although fluctuations over time were observed (e.g., peaks on day 1 and day 13 in the local batch). To complement the interpretation of statistical significance, partial eta squared (η^2^) was calculated according to Equation (3), a measure of effect size that quantifies the proportion of variance in the dependent variable explained by each factor, excluding other effects. Equation (3) uses the *F*-value and degrees of freedom to estimate it using this approximation:

(3) $$\eta_{partial}^{2}=\frac{F*df_{effect}}{F*df_{effect}+df_{error}}$$

where

- $\eta_{partial}^{2}$ is the partial eta squared, indicating the proportion of variance in the dependent variable explained by a given factor, after controlling for other sources of variance in the model;
- ${df}_{effect}$ is the degrees of freedom associated with the factor being analyzed (e.g., Batch, Day, or their interaction);
- ${df}_{error}$ is the degrees of freedom associated with the error or residual term in the model;
- *F* is the *F*-value from the ANOVA table.

This approach allows effect size estimation even when sums of squares are not available. According to Cohen’s guidelines, partial η^2^ values of approximately 0.01 indicate a small effect, 0.06 a medium effect, and 0.14 or higher a large effect. These thresholds provide a practical framework for interpreting the impact of each factor beyond statistical significance alone. According to Table 4, for ammonia, the “Day” factor had a significant effect on ammonia concentration (*p* = 0.001), with a partial η^2^ of 0.440, indicating a moderate to large effect size. “Batch” and the “Batch × Day” interaction were not statistically significant (*p* = 0.18 and *p* = 0.41, respectively), with small effect sizes (η^2^ = 0.071 and η^2^ = 0.108, respectively).

This study revealed distinct respiration patterns between untreated and commercial “Conference pears”, monitored through CO_2_ laser photoacoustic spectroscopy (CO_2_LPAS). Ethylene concentrations peaked earlier in supermarket pears, likely due to post-harvest treatments designed to accelerate or control ripening. Ethanol levels increased progressively, with supermarket pears exhibiting a faster accumulation, suggesting a shift toward fermentation under extended storage. Although initially low, ammonia concentrations showed a marked rise during the later stages of shelf life, particularly after days 8–10, aligning with visible senescence symptoms. The strong correlations observed between ethylene, ethanol, and ammonia concentrations underscore the interconnected metabolic changes occurring during fruit ripening and deterioration. These findings validate the use of CO_2_LPAS as a sensitive, non-invasive tool for real-time quality assessment and propose ammonia as a novel early biomarker for fruit degradation monitoring.

### 2.4. AI-Based Ripening Classification

To complement the chemical analysis of gas emissions, a CNN model was employed to classify pear images into four ripening stages corresponding to 1, 6, 8, and 13 days after harvest. The model was trained and validated using the augmented dataset described in Section 4.4, and its performance was evaluated on a held-out test set.

**Figure 6:**
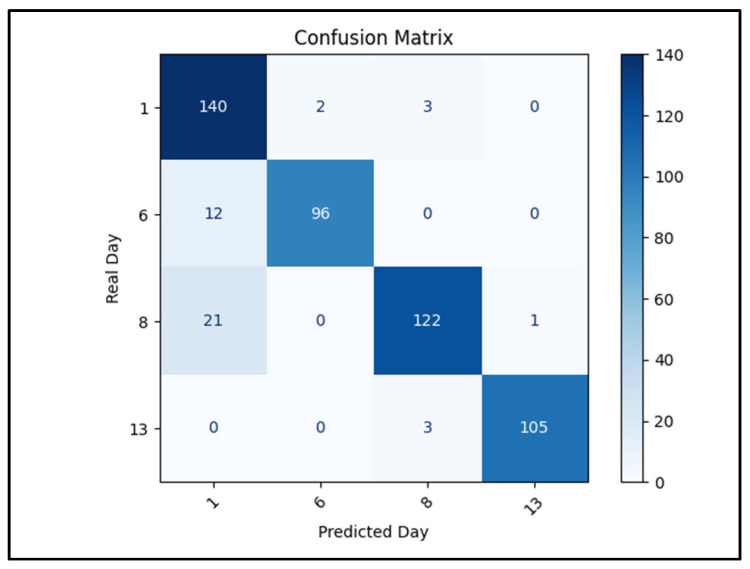
Confusion matrix of the CNN model applied to pear ripening stage classification on the test set (91.68% accuracy). Each cell shows the number of predictions for a given true label (rows) and predicted label (columns). The highest accuracy was achieved for day 1 and day 13 images. Misclassifications were primarily due to day 6 and day 8 samples being incorrectly classified as day 1.

The model achieved an overall classification accuracy of 91.68% on the test set, with a validation accuracy of 96.03% during training. As shown in the confusion matrix (Figure 6), the highest classification rates were achieved for images from day 1 (140/145 correctly classified) and day 13 (105/108), which exhibit clear visual characteristics due to firmness loss or color change.

Most misclassifications occurred with samples from day 8 and day 6, which were sometimes predicted as day 1. Specifically, 21 day 8 samples and 12 day 6 samples were misclassified as day 1, suggesting that early-stage visual features—such as firmness, gloss, or surface texture—may overlap more than expected, leading to confusion. Interestingly, the confusion between day 6 and day 8, which are temporally closer, was minimal.

These results indicate that the CNN model effectively captures ripening cues in visual data and is capable of classifying stages even with subtle inter-class differences. This supports the use of AI-based image classification as a non-invasive, scalable approach to ripeness monitoring, particularly when combined with complementary gas emission data obtained via CO_2_LPAS.

## 3. Discussion

Analyzing the ethylene emission profile shown in Figure 1, it can be seen that higher concentrations were detected in untreated pears with a maximum concentration of 2.157 ± 0.678 ppm (c ± SD) compared to the supermarket batch that had a maximum concentration of 1.738 ± 0.678 ppm (c ± SD), which suggests an increased ripening activity. Higher ethylene production aligns with natural physiological processes in pears, where ethylene acts as a key ripening hormone [28]. Empirical investigations demonstrated a post-harvest surge in ethylene biosynthesis, notably more pronounced under ambient temperature conditions than in cold storage. This heightened ethylene production correlates with accelerated fruit maturation, manifested by rapid chromatic alterations, diminished tissue firmness, and modifications to the fruit’s chemical profile. Specifically, elevated ethylene levels are associated with increased anthocyanin accumulation and accelerated degradation of cell wall polysaccharides, substantially reducing fruit firmness. Moreover, ambient temperature significantly enhances the rate of fruit ripening [29,30,31]. Commercial pears exhibited lower ethylene levels, possibly due to post-harvest treatments designed to delay ripening [32]. Climacteric fruits are prone to rapid spoilage due to an ethylene burst during ripening, leading to softening, mealiness, and increased pathogen susceptibility, which results in high post-harvest losses. The reduction of losses necessitates the regulation of ethylene, a phytohormone that mediates fruit ripening and senescence [33]. The maintenance of freshness, nutritional integrity, palatability, and overall quality of freshly harvested produce requires judicious handling. Given the inherent fragility and perishability of fruits and vegetables, inadequate post-harvest storage can precipitate substantial losses. The domain of post-harvest practices addresses critical aspects of handling, sorting, grading, washing, storage, transportation, and temperature regulation, all of which are indispensable for mitigating produce losses and preserving nutritional content [34]. 1-MCP, an ethylene receptor antagonist, impedes ethylene-mediated ripening and metabolic processes, thus mitigating scalding and prolonging storage. However, it also attenuates fruit ripening. Applying a sequential 1-MCP and ethylene treatment effectively regulates scalding, extends shelf life, minimizes post-harvest decay, and recovers the fruit’s ripening competence [35,36,37]. While genetic techniques like CRISPR/Cas9 are being developed, established post-harvest treatments such as 1-methylcyclopropene (1-MCP), polyamines, salicylic acid, and ozone are widely used [38]. Metal-based catalysts at low temperatures are also being investigated as a safer alternative [39,40,41,42,43,44].

Figure 2 shows that ethanol levels were elevated in commercial pears, likely due to post-harvest treatment effects. Ethanol production is often associated with anaerobic respiration, which may occur when fruit undergoes controlled atmosphere storage or chemical treatment [6,45,46]. This suggests that commercial pears may have been stored under conditions promoting ethanol buildup. Ethanol concentration at the end of the shelf-life storage was 34.406 ± 11.614 ppm from the local batch and 32.612 ± 8.368 ppm from the supermarket. The increased ethanol concentration in pear batches during live storage described a possible shift in energy metabolism from respiration to fermentation [47], as it coincided with the decrease in ethylene concentration.

Minor variations in the ammonia concentration were observed in the respiration of the two batches of pears, as seen in Figure 3, indicating a limited impact on the dynamics of respiration. However, the late-stage increase in ammonia suggests activation of nitrogen metabolism and protein degradation during senescence. The maximum concentration from the local batch was 2.778 ± 0.744 ppm, and from the supermarket batch, 2.451 ± 0.292 ppm was the maximum concentration determined in the pears’ respiration. In this study, ammonia (NH_3_) was investigated as a potential novel biomarker for pear senescence using CO_2_ laser photoacoustic spectroscopy. Our statistical analysis (Table 4) revealed a significant effect of ripening day on ammonia emissions (*p* = 0.001), consistent with physiological changes during fruit maturation and senescence. However, no statistically significant differences were found between batches (*p* = 0.18), nor was there a significant interaction between batch and day (*p* = 0.41). In addition to statistical significance testing, effect size measures were calculated to better understand the magnitude of the observed effects in the ammonia emission data. Partial eta-squared (η^2^) values were determined for each ANOVA factor. The effect of “Day” on ammonia concentration yielded a partial η^2^ of 0.440, indicating a moderate to large effect size and confirming that time significantly influences ammonia dynamics during fruit senescence. In contrast, both the “Batch” factor (η^2^ = 0.071) and the “Batch × Day” interaction (η^2^ = 0.108) showed small effect sizes, supporting the conclusion that storage source (local vs. supermarket) had only a limited influence on ammonia emissions. These findings reinforce the notion that ammonia production is primarily driven by internal physiological changes associated with ripening and senescence rather than external storage conditions. Further studies with larger, more diverse sample sets are necessary to validate ammonia’s robustness as a universal biomarker for fruit senescence. Previous studies also indicated ammonia’s role as a senescence-related volatile compound in fruits and plants [48]. Ammonia production in plant tissues is typically associated with protein degradation and nitrogen metabolism, processes that intensify during cellular breakdown in advanced ripening stages [49,50]. While ammonia detection in fruit respiration is less common than ethylene or ethanol, recent evidence suggests its presence signifies specific physiological [51] or microbial degradation processes [52]. For instance, photoacoustic [53] and GC-MS spectroscopy studies [54] were used to detect ammonia levels in fruits. Pears [55,56] and apples [57,58,59] have observed elevated NH_3_ concentrations during extended storage or in response to microbial decay, particularly under conditions of hypoxia or stress. Analogously, in bananas [60,61,62,63], ammonia emissions are correlated with advanced senescence stages, plausibly due to intensified amino acid catabolism. These observations corroborate the hypothesis that NH_3_ does not function as a generic ripening marker; instead, it emerges as a byproduct of nitrogen metabolism intrinsically linked to protein breakdown [61]. The convergent trends documented across pears, apples, and bananas collectively indicate that ammonia could serve as a late-stage biomarker of fruit quality deterioration, especially under suboptimal storage conditions or after shelf life. Consequently, its integration into real-time monitoring frameworks holds the potential to augment the early detection of spoilage, extending beyond conventional ethylene-based assessment. We emphasize that the present findings serve as an initial step toward identifying ammonia as a senescence marker, highlighting the need for expanded experiments to confirm and extend these observations.

The respiration rate of fruits is a function of multiple variables, including fruit type, maturity stage, size, age/storage duration, microbial infection, and physical wounding. Cultivar-specific differences in respiration are observed across commodity types. Fruit maturity positively correlates with respiration rate, while fruit size demonstrates an inverse relationship. Senescence and microbial infection are associated with increased respiration. Physical wounding induces ethylene biosynthesis, resulting in a significant elevation of respiration rate [64,65]. The differences in gas emissions between local and commercial pears highlight the impact of storage and treatment conditions. Our findings align with previous research on fruit respiration, demonstrating PAS as a reliable method for gas detection. Specifically, similar trends in ethylene suppression due to commercial treatments were observed in apples and bananas, supporting the hypothesis that industrial storage conditions alter natural respiration patterns [66]. Ethylene production is a well-known trigger of climacteric fruit ripening, promoting cell wall softening and pigment changes. Ethanol accumulation reflects a partial shift toward anaerobic respiration, often induced under storage stresses. The observed rise in ammonia is consistent with protein degradation pathways activated during metabolic processes. Adding ammonia monitoring to traditional ethylene analysis may enable earlier detection of fruit quality decline, allowing for optimized storage strategies and reduced post-harvest losses.

In addition to the spectroscopic methods used for real-time monitoring of volatile gases, the integration of an AI model provided a complementary approach for the non-invasive assessment of fruit ripening. The use of CNNs significantly contributed to the visual classification of ripening stages, achieving an accuracy of 90.59% even under limited sampling conditions. This performance validates the potential of CNNs to extract subtle visual features associated with physiological changes in the fruit, which often precede visible signs of degradation.

Furthermore, correlating the classifications made by the CNN model with gas dynamics measured through CO_2_LPAS, particularly ethylene and ammonia, revealed a temporal alignment between the visual predictions of ripening stages and the metabolic changes detectable in volatile emissions. For example, the stage identified by the network as “day 8” coincided with the onset of accelerated ammonia accumulation, a biochemical marker of advanced senescence, supporting the AI’s potential to indicate or predict key physiological events. This allowed the classification of test images into discrete post-harvest aging categories, providing a visual-ripening profile that could be correlated with the physiological gas emission data recorded by the CO_2_LPAS system.

This study demonstrated that alongside traditional markers such as ethylene and ethanol, ammonia emission dynamics can provide valuable early indicators of “Conference” pear senescence. The use of CO_2_LPAS enabled real-time, non-invasive detection of volatile compounds with high sensitivity, offering a practical tool for monitoring post-harvest fruit physiology. From a physiological perspective, the observed gas emission patterns align with established ripening and deterioration processes in climacteric fruits. Ethylene production triggered ripening-associated changes such as tissue softening and pigment accumulation, while progressive ethanol accumulation suggested a metabolic shift toward anaerobic respiration under shelf-life conditions. The significant late-stage rise in ammonia concentration is consistent with protein degradation and intensified nitrogen metabolism, processes typically associated with advanced stages of senescence. This pattern suggests that ammonia could serve as a novel biomarker for assessing the transition from ripening to irreversible quality decline in pears. These findings have important practical implications. Integrating ammonia monitoring alongside ethylene and ethanol measurements could improve the early detection of fruit spoilage, allowing for timely interventions in storage and distribution systems. Recent developments in photoacoustic spectroscopy for trace gas detection demonstrated the importance of laser wavelength stability, power control, and system design in achieving ppb-level sensitivity. Yin et al. developed a highly sensitive photoacoustic sensor system that integrates a high-power quantum cascade laser (QCL) for sulfur dioxide (SO_2_) detection, achieving a detection limit of 2.45 ppb [67]. According to the abstract by Chen et al. [68], their study presents the development of a trace-level photoacoustic SO_2_ gas sensor designed to monitor SO_2_ decomposition products in SF_6_ gas. The system employs a 266 nm UV laser along with an acousto-optic power stabilizer to address the challenge of laser power instability in the UV region. This design enables a high quality factor (Q-factor) and achieves a detection limit of 140 ppbv. Additionally, Yin et al. [69] reported a QEPAS-based sensor for detecting sub-ppm levels of H_2_O in an SF_6_ buffer gas. Their system reached a detection limit of 0.49 ppm with a 1 s averaging time, demonstrating its potential as a robust tool for safety monitoring in power systems. These approaches underline the significance of optimizing laser parameters, such as those implemented in our CO_2_LPAS setup for ethylene, ethanol, and ammonia detection. The non-invasive, real-time capability of CO_2_LPAS further enhances its potential for deployment in commercial post-harvest management, offering a more precise, automated approach to optimizing fruit shelf life and reducing food losses. However, it should be noted that this study focused on a single pear cultivar under specific storage conditions. Although our results suggest that the lower ethylene concentrations observed in supermarket pears may be due to post-harvest treatments such as 1-MCP, we did not have access to treatment metadata or perform residue analysis to verify this assumption. This represents a limitation of the current study and should be addressed in future work through direct chemical characterization of potential treatment agents. Broader validation across different cultivars, storage environments, and stress conditions is necessary to confirm the general applicability of ammonia as a universal marker of fruit senescence. Additionally, larger sample sizes and long-term studies would strengthen the robustness of these findings. Future research will aim to expand this methodology to a wider range of climacteric and non-climacteric fruits, investigate the combined dynamics of multiple volatile compounds, and explore the integration of CO_2_LPAS into smart storage systems equipped with automated monitoring and decision-making capabilities.

## 4. Materials and Methods

### 4.1. Sample Preparation

This study analyzed two batches of Conference pears: one sourced from the Experimental Field of the Faculty of Horticulture within the University of Agronomic Sciences and Veterinary Medicine of Bucharest (referred to as the local batch) and another batch purchased from a supermarket, potentially subjected to post-harvest treatments. The pears were stored under controlled conditions, kept at room temperature (20 ± 1 °C) and 80 ± 5% relative humidity, to simulate typical shelf-life conditions. The local batch exhibited a lower average weight of 92.66 g, while the supermarket batch had a higher average of 138 g. Each batch consisted of 30 pears, and each data point represents the mean of three biological replicates (*n* = 3), measured on different individual pears. For each measurement, individual pears were placed into a glass cuvette with a volume of 150 cm^3^ at room temperature and then connected to the CO_2_LPAS system for analysis. Gas concentrations were recorded over a 13-day shelf-life period for the local pear batch and over a 10-day shelf-life period for the supermarket pear batch, enabling high-resolution temporal tracking of ethylene, ethanol, and ammonia emission profiles for each pear batch.

### 4.2. CO_2_LPAS Measurement System

Respiration measurements were conducted using a CO_2_ laser-based PAS system. The setup included a photoacoustic cell equipped with a CO_2_ laser source for selective gas detection and calibration with standard gas mixtures to ensure measurement accuracy. Gas concentrations were recorded at fixed time intervals, and statistical analyses were performed to assess significant differences [70,71,72].

The primary goal of this system was to provide accurate, non-invasive, and efficient monitoring of gas concentrations—specifically ethylene, ammonia, and ethanol—during the post-harvest fruit respiration and to investigate plant physiology using CO_2_LPAS. This multi-component CO_2_LPAS system allows for the simultaneous, real-time detection of trace gases in the parts-per-billion (ppb) range with high sensitivity and selectivity. Using a CO_2_ laser ensures adequate output power for precisely detecting these gases, enabling timely and effective interventions to prevent fruit spoilage. The system employs a CO_2_ laser tuned to ethylene, ethanol, and ammonia absorption lines alongside a high-sensitivity photoacoustic cell. Gas samples were collected periodically and analyzed in real time.

**Figure 7:**
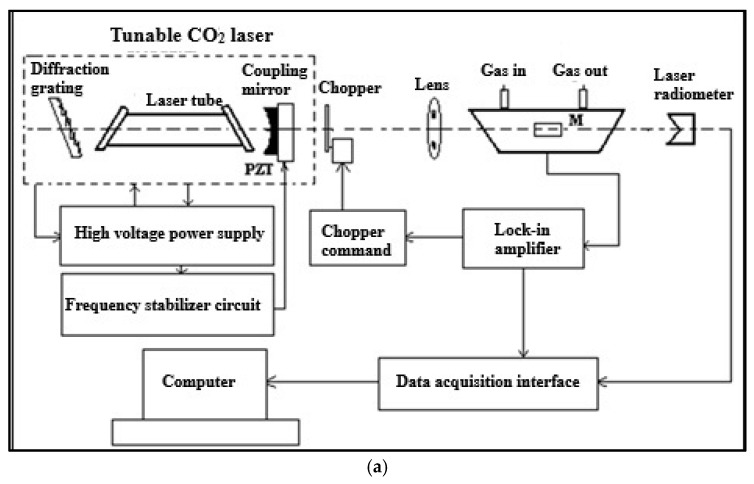

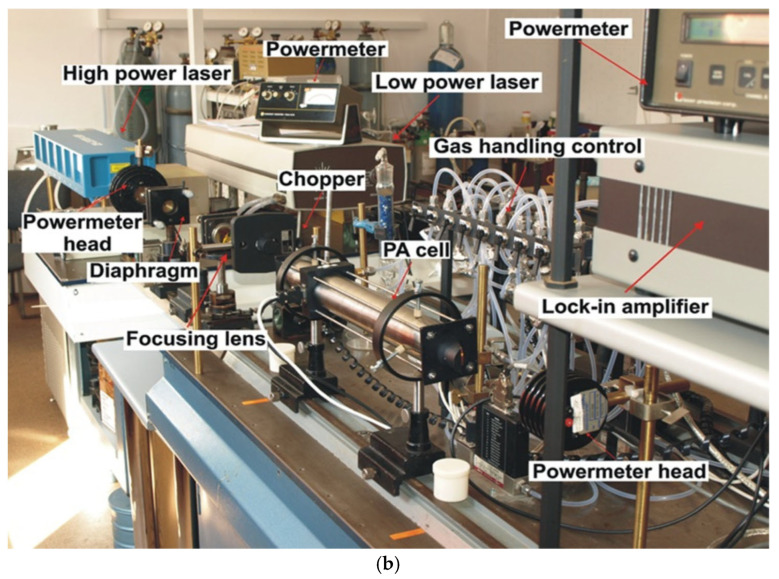
(**a**) Schematic diagram of the CO_2_ laser photoacoustic spectroscopy (CO_2_LPAS) system. (**b**) Photograph of the complete laboratory setup used for gas detection experiments.

The experimental setup consisted of a laser source, a photoacoustic cell, detection, data acquisition systems, and a gas handling system (see Figure 7). The radiation source was a custom-built CO_2_ laser that emits a stabilized, tunable continuous wave (cw) from 9.2 µm to 10.8 µm across 57 spectral lines corresponding to vibrational–rotational transitions grouped into four branches: 9R, 9P, 10R, and 10P [70]. The CO_2_ laser employed in this study operates across a tunable range of 9.2–10.8 µm (corresponding to the 9R, 9P, 10R, and 10P branches), allowing selective excitation of characteristic absorption lines for each target gas. The following laser lines were used for optimized detection: C_2_H_4_: 10P(14) [70] at 10.53 µm; C_2_H_6_O: 10P(20) at 10.34 µm [72]; and NH_3_: 9R(30) at 9.22 µm [73]. The output power ranged between 1 and 5 W, depending on the line, and was continuously monitored using a calibrated radiometer (Laser Probe RKT 30 CAL (Laser Probe, Inc., Utica, NY, USA)) to ensure accurate signal normalization. The detection sensitivity of the photoacoustic system was directly proportional to the incident laser power and the absorption coefficient at the selected wavelength. These lines were chosen based on published absorption cross-section data and our prior calibration measurements [74,75]. The gases detected by the system must have high absorption coefficients in the CO_2_ laser’s wavelength range. Inside the photoacoustic (PA) cell, gas traces absorb the laser radiation, converting the absorbed energy into heat, which increases the pressure in the closed volume. The cw laser radiation is amplitude-modulated by a mechanical modulator (DigiRad C-980 (DigiRad Corporation, Poway, CA, USA)), operating at the same frequency (564 Hz) as the PA cell. A lens focuses the radiation before entering the PA cell, where the target gas molecules absorb a small fraction. The laser power within the PA cell is considered constant, with the radiometer (Laser Probe RKT 30 CAL) providing the real power value inside the cell.

Four miniature electret microphones (Knowles EK-303 or EK-23024 (Knowles Corporation, Itasca, IL, USA)) were connected in series to detect acoustic waves produced in the PA cell. The PA signal is directly proportional to the gas concentration. The microphone signals were amplified by a Stanford Research Systems model SR 830 dual-phase lock-in amplifier, which synchronizes the signal with the modulator’s phase. This signal was automatically recorded in real time by a National Instruments acquisition board (NI cDAQ-9174 (National Instruments (NI), Austin, TX, USA)) controlled by a computer. Both the amplifier and radiometer output signals were converted into digital form via a high-speed 12-bit A/D board for processing.

The PA signal, which is proportional to the number of absorbing molecules in the PA cell, serves as an indicator of gas concentration. The system was designed to produce no signal in the absence of target molecules in the gas mixture, adhering to a “zero-base” principle. At a signal-to-noise ratio (SNR) of 1, the minimum measurable signal (*V*_min_) corresponds to the minimum detectable concentration (*c*_min_), which can be calculated as

$$c_{min}=V_{min}/P_{L}R$$

To address outgassing issues, a stainless steel PA cell with a volume of 1 dm^3^ was employed. The cell’s design, which includes dimensions (300 mm length, 7 mm inner diameter), an acoustic resonator tube, windows, gas inlets/outlets, microphones, and an acoustic filter, was optimized for optimal signal performance. The quality factor (Q) of the PA cell was 16.1, with a cell constant (C) of 4375 Pa cm/W and a responsivity (R) of 350 cmV/W, indicating the sensitivity of the cell to pressure changes [70].

The gas handling system was designed to ensure reproducibility in dynamic and static regimes, controlling gas introduction, evacuation, partial pressures, flow rates, and pressure gradients for various gas components. This system also facilitates the controlled elimination of residual gas components and provides access to biological samples or gas sample bags. To improve the accuracy of PA signal measurements, a potassium hydroxide (KOH) scrubber was placed between the sampling cell and the PA cell to reduce atmospheric interference from water vapor and carbon dioxide [76]. After each measurement, the system underwent a thorough cleaning cycle with nitrogen (6.0 grade, 99.9999% purity) to ensure measurement integrity, with the background signal capped at 35 µV for a well-cleaned system [77].

The gas supply system for a measurement cell, for static and dynamic gas mixtures at set concentrations, ensures the following: gas introduction/evacuation; partial/total gas pressure control; flow rate/pressure gradient control; gas exhaust; residual gas removal (CO_2_, H_2_O_2_, etc.); cell wall residue removal; biological/exhaled air sample access; and sealed bag gas sampling.

Samples were connected to the PA cell via a sealed system. Glass cuvettes or desiccators were used, and a Baratron 122A manometer monitored pressure. Gas flow monitoring raises the issue of source vs. measured concentration due to potential reactions/adsorption. Polar molecules pose adsorption challenges, mitigated by material selection. The vacuum/gas system is vital for cell/gas purity. Its functions include cell pumping, gas mixing, and pressure monitoring. Gas flow minimizes vapor adhesion and background noise. An effective system should perform the following: vacuum–evacuate the system/cell; control gas/mixture input (N_2_ rinse, calibration); measure cell/system pressure; safely introduce sample cuvettes/bags; filter interfering gases (CO_2_, H_2_O); control trace gas input with a carrier gas (N_2_, synthetic air); adjust sample/carrier flow (10–1000 sccm (standard cubic centimeter by minute)); simultaneously measure multiple gases; and provide rapid (seconds/minutes) gas concentration monitoring.

### 4.3. Data Analysis

Gas concentration variations were analyzed using statistical methods, including ANOVA, to determine significant differences between the two pear batches. Correlation coefficients were computed to evaluate relationships between the three gases over time.

### 4.4. AI-Based Image Analysis

In parallel with gas concentration measurements, a CNN model was developed to estimate the ripening stage of pears based on visual cues. The CNN was trained using grayscale images of pears categorized by post-harvest age (1, 6, 8, and 13 days after harvest). The images were stored in a structured folder hierarchy, and preprocessing included grayscale conversion, resizing to 128 × 128 pixels, normalization, and reshaping for model compatibility.

To enrich the dataset and reduce overfitting risk, we applied systematic data augmentation. Each image was rotated from 0° to 300° in 60° steps, and for each rotation, a horizontal flip (left–right) was applied. This procedure generated multiple transformed versions of each input image, simulating different orientations and viewing angles that can occur in real-world monitoring setups.

The augmented dataset was then split as follows: 10% was held out as a test set, while the remaining 90% was split into 81% training and 9% validation sets. This resulted in final proportions of 81% training, 9% validation, and 10% test data. Stratified sampling was used to ensure a balanced representation of each ripening stage across all subsets.

**Figure 8:**
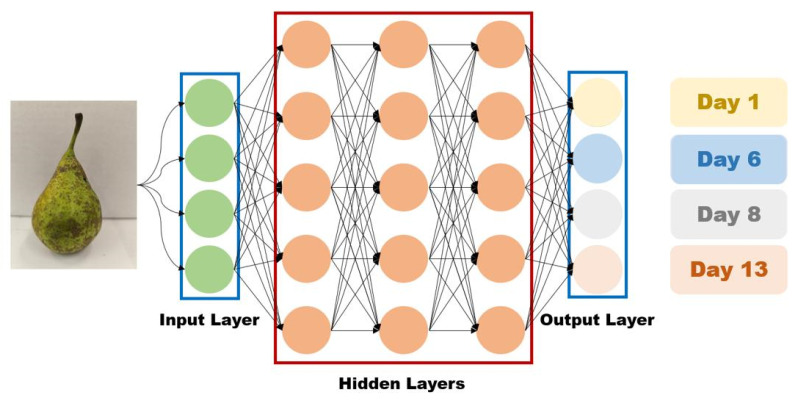
CNN model for estimating pear age: 1, 6, 8, and 13 days based on images.

The CNN architecture comprised four convolutional layers [78] with Rectified Linear Unit (ReLU) activations [79] and max-pooling [80] followed by a fully connected layer [81] and dropout regularization (rate = 0.2) [82]. The final classification layer used a sigmoid activation function [83] to produce class probabilities corresponding to the number of ripening stages. The model was trained using the Adam optimizer [84] and sparse categorical cross-entropy loss for 20 epochs with a batch size of 32 (Figure 8).

The detailed implementation of the CNN architecture and training pipeline is available online and supports the analysis presented [85]. A dedicated Python (version 3.12.4) pipeline was also implemented to automate batch prediction and evaluation across all subsets.

As this study focuses on a single pear cultivar (Conference), all image batches refer to the same source material. Thus, the AI-based analyses refer to images obtained from a single unified experimental batch. Nonetheless, this approach provides a non-invasive, scalable method that could be adapted to other fruit types or sensor-integrated quality control systems.

## 5. Conclusions

This study demonstrates the effectiveness of CO_2_ laser photoacoustic spectroscopy (CO_2_LPAS) as a sensitive, non-invasive method for monitoring the post-harvest respiration of Conference pears. By simultaneously detecting ethylene, ethanol, and ammonia emissions, CO_2_LPAS provided valuable insights into the physiological processes underlying fruit ripening and senescence.

The results highlight significant differences in volatile gas dynamics between untreated and commercially stored pears, with early ethylene peaks and accelerated ethanol accumulation observed in supermarket batches. Notably, the late-stage rise in ammonia concentrations suggests its potential role as a novel early biomarker for fruit quality decline—a finding that offers new perspectives for post-harvest management and shelf-life prediction.

The integration of real-time gas monitoring into storage and distribution systems could enable more proactive strategies to maintain fruit freshness, reduce waste, and optimize supply chain logistics. Future work will focus on validating ammonia as a universal marker across different cultivars and storage conditions and on further developing CO_2_LPAS systems for automated, high-throughput applications in the agri-food industry.

In parallel, AI (particularly CNNs) has shown strong potential in non-invasive visual classification of ripening stages. When combined with gas emission data, this hybrid approach offers enhanced accuracy for real-time quality monitoring and decision-making in post-harvest management.

This work lays the foundation for advancing non-invasive technologies that can transform post-harvest quality control and support a more sustainable, efficient food supply chain. While the results are promising, certain limitations should be considered when interpreting the results. The relatively small sample size and lack of metadata on post-harvest treatments for supermarket pears limit the generalizability of some findings. Additionally, the CNN model was trained on a single pear cultivar, which may restrict its applicability to other fruit types or conditions. Future research should address these aspects by expanding the dataset, including detailed storage metadata, and validating the AI model across broader scenarios.

In future work, the developed CO_2_ laser photoacoustic spectroscopy system will be applied to monitor the respiration of other climacteric and non-climacteric fruits. Expanding the range of detected volatile compounds, including potential early stress markers, will be pursued. Furthermore, integrating this gas monitoring technology into smart storage systems could enhance post-harvest quality control and contribute to reducing food loss.

## Figures and Tables

**Table 1:** Pearson correlation: gas relationships.

Pair	*r*-Value	*p*-Value
C_2_H_4_ vs. C_2_H_6_O	0.82	<0.001
C_2_H_4_ vs. NH_3_	0.65	0.003
C_2_H_6_O vs. NH_3_	0.71	<0.001

**Table 2:** Two-way ANOVA analysis for ethylene (C_2_H_4_).

Factor	*F*-Value	*p*-Value	Conclusion (α = 0.05)
Batch	15.2	<0.001	Reject H_0_
Day	28.7	<0.001	Reject H_0_
Batch × Day	3.1	0.02	Interaction

**Table 3:** Two-way ANOVA analysis for ethanol (C_2_H_6_O).

Factor	*F*-Value	*p*-Value	Conclusion
Batch	25.41	<0.001	Significant
Day	38.76	<0.001	Significant
Interaction	7.92	0.002	Significant

**Table 4:** Two-way ANOVA analysis for ammonia (NH_3_).

Factor	*F*-Value	*p*-Value	Conclusion	Partial η^2^
Batch	1.84	0.18	Not significant	0.071
Day	6.29	0.001	Significant	0.440
Batch × Day	0.97	0.41	Not significant	0.108

## Data Availability

The authors confirm that all data are available within the article.
